# The impact of induced anxiety on affective response inhibition

**DOI:** 10.1098/rsos.170084

**Published:** 2017-06-07

**Authors:** Jessica Aylward, Vincent Valton, Franziska Goer, Anahit Mkrtchian, Níall Lally, Sarah Peters, Tarun Limbachya, Oliver J. Robinson

**Affiliations:** Institute of Cognitive Neuroscience, University College London, 17–19 Queen Square, London WC1N 3AZ, UK

**Keywords:** threat of shock, affective response inhibition, stress, mood and anxiety disorders

## Abstract

Studying the effects of experimentally induced anxiety in healthy volunteers may increase our understanding of the mechanisms underpinning anxiety disorders. Experimentally induced stress (via threat of unpredictable shock) improves accuracy at withholding a response on the sustained attention to response task (SART), and in separate studies improves accuracy to classify fearful faces, creating an affective bias. Integrating these findings, participants at two public science engagement events (*n* = 46, *n* = 55) were recruited to explore the effects of experimentally induced stress on an affective version of the SART. We hypothesized that we would see an improved accuracy at withholding a response to affectively congruent stimuli (i.e. increased accuracy at withholding a response to fearful ‘no-go’ distractors) under threat of shock. Induced anxiety slowed reaction time, and at the second event quicker responses were made to fearful stimuli. However, we did not observe improved inhibition overall during induced anxiety, and there was no evidence to suggest an interaction between induced anxiety and stimulus valence on response accuracy. Indeed Bayesian analysis provided decisive evidence against this hypothesis. We suggest that the presence of emotional stimuli might make the safe condition more anxiogenic, reducing the differential between conditions and knocking out any threat-potentiated improvement.

## 1. Introduction

Anxiety can be both adaptive and maladaptive, but in both cases lead to altered cognitive performance [1] Anxiety can, for instance, promote a tendency to focus on negative stimuli at the expense of positive ones; a so-called ‘negative bias’ [1]. While a bias towards these stimuli can be adaptive, encouraging the adoption of behaviours that reduce risk of harm, it may also precipitate the onset of mood disorders [2,3]. Indeed, negative biases in cognition are prevalent in people suffering from anxiety disorders [4,5] and major depressive disorder [6,7]. One hypothesis, therefore, is that pathological anxiety is an extension of the same mechanisms that contribute to adaptive anxiety [8].

Inducing adaptive anxiety in healthy controls may thus enable the interaction between anxiety and cognition to be investigated, contributing to our understanding of the mechanisms that underlie maladaptive mood states. A state of anxiety, thought to be related to pathological anxiety [8] can be induced in participants by introducing the threat of an unpredictable electric shock, a well-validated technique [9,10]. In a within-subjects design, the influence of anxiety on cognition can be investigated by comparing task performance in the same individual when they are at risk of an unpredictable electric shock and when safe from shock. This technique has previously been used to investigate the interaction between anxiety and response inhibition—the ability to withhold a response—using a sustained attention to response task (SART). In this task, participants must respond to frequent ‘go’ target stimuli, and withhold a response to infrequent ‘no-go’ distractor stimuli. Threat of shock increases accuracy to ‘no-go’ distractor stimuli [11,12] while slowing down response time overall [9,13].

Threat of shock can also instantiate a negative affective bias: enhancing the processing of affectively congruent stimuli [10,14]. When at risk of shock, response accuracy is improved and acoustic startle reactivity is increased to unexpected fearful faces, relative to unexpected happy faces [15,16]. Taken together these findings indicate that threat of shock may promote harm-avoidant behaviour [11].

Threat of shock can therefore selectively enhance processing of affectively congruent stimuli and promote inhibitory control. In this study, conducted over two public scientific engagement events and a follow-up laboratory sample, we used an adapted version of the SART to investigate whether inhibitory effects interact with affective bias. In this new task, participants were instructed to respond to happy faces and withhold responses to fearful faces (and vice versa) whilst they were alternately at risk and safe from unpredictable shock. We predicted that the threat of shock manipulation would induce a state of transient anxiety in participants, and we would see an improvement in accuracy to withhold a response to affectively congruent stimuli (i.e. increased accuracy at withholding a response to fearful ‘no-go’ distractors) under threat of shock. The nature of the scientific engagement events also enabled us to investigate effects within a more naturalistic setting than in highly controlled laboratory conditions.

## 2. General methods

This study was completed as part of two public engagement events: the first at the Royal Institution, entitled ‘Questioning Reality’^1^ and a second at the Wellcome Collection, entitled ‘Feeling Emotional’^2^. We also ran a follow-up study at UCL. At the engagement events, data were collected in a large room, with two computers set up, but event attendees were free to wander around the room and watch the testing. The overall sound levels and potential for distraction were considerably greater than a controlled testing environment but can be thought of as a naturalistic representation of an informal social event. All participants provided written informed consent (UCL ethics reference: 1764/001) and completed a screening form that verified they had no history of neurological, psychiatric or cardiovascular conditions.

### 2.1. Stress manipulation

Two electrodes were attached to the back of the participants' wrists (on their non-dominant hand) to induce stress via threat of unpredictable electric shock. They were instructed to make their responses using the opposite hand. For each participant, the shock level was set to a level where it was ‘unpleasant but not painful’ [17]. Shocks were delivered using a Digitimer DS5 Constant Current Stimulator (Digitimer Ltd, Welwyn Garden City, UK).

### 2.2. Stress manipulation check

Immediately after completing the task participants were asked to report how anxious, afraid and stressed they had felt in the safe and threat conditions. They made a response between 1 (not at all) and 10 (very much so).

### 2.3. Power analysis

An *a priori* power analysis was run in G*Power [18]. The power analysis was based on previous results of the SART [11] that gave an effect size of 0.56 for the effect of threat of shock on response accuracy to ‘no-go’ distractor stimuli. A power calculation determined that we needed 44 participants with 95% power (with alpha 0.05, two tailed) to detect an effect size of 0.56. Owing to the popularity of the events, we collected data from an extra 3 and 12 participants at each event.

### 2.4. Affective response inhibition task

**Figure 1.**
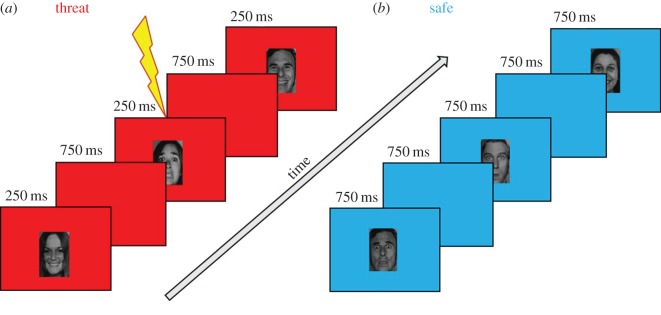
Participants were instructed to press the space bar as quickly as possible for target, ‘go’ stimuli and withhold responses to infrequent distractor, ‘no-go’ stimuli (‘go’ and ‘no-go’ stimuli valence was counterbalanced). (*a*) Study 1 represented. Participants received an unpredictable electric shock (independent of behavioural response) during the threat condition. (*b*) Study 2 represented. Participants were not at risk of shock during the safe condition.

Participants completed a new task adapted from our previously used SART [11] under two different conditions; they were alternately at risk of an unpredictable shock and safe from a shock. For 2 s at the beginning of each block, ‘YOU ARE NOW SAFE FROM SHOCK!’ or ‘YOU ARE NOW AT RISK OF SHOCK!’ (order counterbalanced), appeared on the screen. When in a safe block, the background was blue (and no shocks were delivered), and when in a threat block, the background was red (figure 1). Participants were required to respond to frequent ‘go’ target stimuli by pressing the space bar as quickly as possible after presentation and to inhibit a response to infrequent ‘no-go’ distractor stimuli by withholding a key press. In keeping with the original study [11], in order to set up a pre-potent response, the ‘go’ to ‘no-go’ stimuli ratio was approximately 10 : 1. For half of the trials, happy faces were the ‘go’ target stimuli, and fearful faces were ‘no-go’ distractor stimuli (order counterbalanced). We chose to use only happy and fearful faces in order to keep our study comparable with previous research [10]. In addition, the exclusion of neutral faces, which are potentially less arousing or salient than fearful and happy faces, increases the possibility that any resulting effects could be explained by valence. Moreover, there is large variation in the perception of face stimuli, which could manifest in neutral face stimuli being perceived as negative [19,20]. Halfway through, the contingency changed (with explicit instructions), and happy faces became ‘no-go’ distractor stimuli, and fearful faces became ‘go’ target stimuli. The order of contingencies was counterbalanced across participants as well as the order of threat and safe blocks. These details are common to both events, but the precise stimuli presentation details differed (see study specific details below).

### 2.5. Study 1

#### 2.5.1. Participants

Forty-seven subjects completed the study. One subject was removed due to inattention (mean target accuracy = 0.02). In total, 46 participants (male = 24) aged between 19 and 65 (*M* = 30.48, s.d. = 9.38) were included in the analysis.

#### 2.5.2. Stimuli details

There were a total of four blocks (two threat and two safe blocks), with each block lasting 52 s. Participants received either one shock (45th threat trial) or two shocks (45th and 97th threat trial), during the shock condition. They were informed that the shocks would be delivered at random and were uncontrollable (i.e. they were not punished as a result of an incorrect key press). Forty-seven ‘go’ stimuli and five ‘no-go’ stimuli were randomly presented in each block. Face stimuli were presented for 250 ms, followed by an interstimulus interval of 750 ms, before presentation of the next stimulus (figure 1*a*). These durations are the same as in the original design [11], but there were a smaller number of blocks, in order to reduce task length. Task duration was 3 min 36 s.

#### 2.5.3. Between-subject measures

Thirty-two participants also completed self-report measures of Trait Anxiety [21]. Blood alcohol concentrations were collected from 23 participants, recorded by asking participants to breathe into an AlcoDigital ProPack 7000 machine. However, it should be noted that we have concerns that the breathalyser was not functioning correctly (e.g. some very high readings were gained alongside self-reported claims that they had not consumed any alcohol for 20 years).

### 2.6. Study 2

#### 2.6.1. Participants

Fifty-six participants (Female = 48) aged between 18 and 60 (*M* = 30.80, s.d. = 9.33) completed the study. One participant was removed due to an absence of responses throughout the task. In total, 55 participants (female = 47) aged between 18 and 60 (*M* = 30.89, s.d. = 9.32) were included in the analysis.

#### 2.6.2. Stimuli details

This was the same as above, with the exception that each block lasted 60 s. There were also fewer stimuli in each block: 36 ‘go’ stimuli (35 in third block) and four ‘no-go’ stimuli (five in third block) were randomly presented. As above, participants received either one shock (this time after the 35th threat trial) or two shocks (this time after the 35th and 60th threat trial). In contrast to Study 1, face stimuli were presented for 750 ms, while the interstimulus interval remained the same at 750 ms (figure 1*b*). The stimulus presentations were longer in this task to ensure that the duration was sufficient to allow for the processing of the emotion. As in Study 1, the number of blocks was fewer than in the original design [11] in order to reduce total task duration. Total task duration was approximately 4 min and 8 s.

### 2.7. Laboratory data

We collected data from a further 18 participants at UCL. The task design remains exactly the same as described under ‘Study 2’ with the exception of the background colour. During safe conditions, the background was red, and during threat conditions the background was blue. These extra data were collected to check whether effects might be driven by colour rather than condition.

### 2.8. Data analysis

Reaction time (RT) and accuracy was analysed using two repeated-measures general linear models in SPSS version 22 (IBM Crop, Armonk, NY). For all analyses, *p* = 0.05, was considered significant. Bayesian statistics were also run for these frequentist analyses (JASP, v. 0.7 [22]), employing the default prior. The Bayesian approach considers the likelihood of data if the alternative if the alternative hypothesis is true versus if the null hypothesis is true, and is a very suitable approach for this research. Data are available for download (http://dx.doi.org/10.6084/m9.figshare.1609723).

Performance accuracy for each condition (threat/safe) and trial type (‘go’/‘no-go’) was calculated by dividing the number of correct trials by the total number of trials.

Accuracy analysis was performed on ‘no-go’ trials only (‘go’ accuracy was 95.2% and 97.1% at the first and second event, respectively). Response accuracy on ‘no-go’ trials was analysed using a two-way ANOVA with factors valence (happy/fearful) and condition (threat/safe). RT analysis was performed on ‘go’ stimuli only (as the only RTs on ‘no-go’ trials are error trials). Prior to analysis, a log transform was applied to RT data as they were not normally distributed. RT to ‘go’ stimuli was analysed using a two-way ANOVA with factors valence (happy/fear) and condition (threat/safe). Bayesian ANOVAs were used to generate BF_10_ factors for models of interest relative to a null model (main effect of subject). The ‘winning’ model BF_10_ was defined as the highest BF_10_ relative to the null. The following labels were assigned to BF_10_: anecdotal (1–3), substantial (3–10), strong (10–30) decisive (more than 100) to interpret the magnitude differences between models [23].

Post hoc analyses were run to investigate the effect of STAI trait anxiety scores and blood alcohol concentration on performance. Pearson's *r* correlations between symptom measures (STAI Trait scores, and blood alcohol concentration) and RT/accuracy data were run. Only *n* = 28 participants could be included in the secondary analysis of STAI anxiety scores in Study 1 due to missing data. All participants' scores (*n* = 55) were included in the secondary analysis of STAI anxiety scores in Study 2.

## 3. Results

### 3.1. Study 1

#### 3.1.1. Threat of shock manipulation

Subjective ratings of anxiety levels were significantly higher during the threat condition relative to the safe condition (*t*_42_ = 5.31, *p* < 0.001, *d* = 0.73; safe *M* = 3.37, s.d. = 2.21, threat *M* = 5.19, s.d. = 2.75). Bayesian analyses confirmed a model with main effect of condition was the winning model, with a decisive magnitude difference relative to the null (BF_10_ = 4816).

#### 3.1.2. Accuracy

We did not find a significant main effect of condition (*F*_1,45_ = 0.005, *p* = 0.95, $\;\eta_{p}^{2}<0.001$), or valence (*F*_1,45_ = 1.06, *p* = 0.31, $\;\eta_{p}^{2}=0.02$) nor was there a significant (valence × condition) interaction in participants' accuracy (*F*_1,45_ = 0.47, *p* = 0.50, $\eta_{p}^{2}=0.01$; see table 1 for response accuracy to each stimulus and trial type).

**Table 1.** Response accuracy and RT to each stimulus and trial type in Study 1.

	mean	s.d.
*response accuracy*		
fearful ‘no-go’ distractors (threat condition)	0.76	0.24
fearful ‘no-go’ distractors (safe condition)	0.74	0.24
happy ‘no-go’ distractors (threat condition)	0.74	0.24
happy ‘no-go’ distractors (safe condition)	0.73	0.24
fearful ‘go’ targets (threat condition)	0.87	0.067
fearful ‘go’ targets (safe condition)	0.97	0.078
happy ‘go’ targets (threat condition)	0.98	0.023
happy ‘go’ targets (safe condition)	0.99	0.016
*mean RT* (*ms*)		
fearful ‘no-go’ distractors (threat condition)	199.18	172.61
fearful ‘no-go’ distractors (safe condition)	196.38	153.73
happy ‘no-go’ distractors (threat condition)	225.25	163.66
happy ‘no-go’ distractors (safe condition)	224.56	205.18
fearful ‘go’ targets (threat condition)	396.45	83.42
fearful ‘go’ targets (safe condition)	376.94	65.78
happy ‘go’ targets (threat condition)	378.14	61.27
happy ‘go’ targets (safe condition)	364.09	51.24

Bayesian analysis confirmed that no models had a BF_10_ > 1 indicating no evidence of an effect of valence or threat. Indeed, a model including an interaction term between valence and threat was substantially (83.3) worse than the null enabling us to reject our hypothesis of a threat × valence interaction.

#### 3.1.3. Reaction time

**Figure 2.**
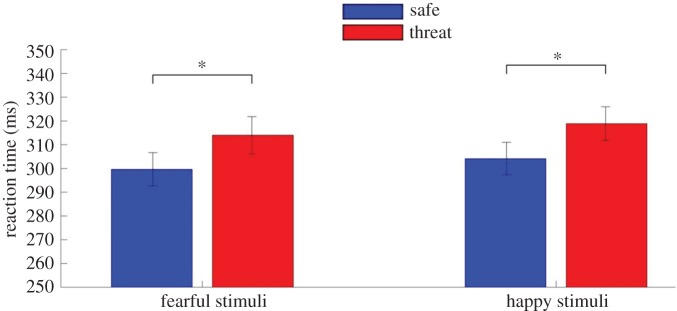
Study 1. Participants were significantly slower to respond to targets (‘go’ stimuli) when under threat of shock (*p* < 0.001). Error bars represent one s.e.m.

We found a significant effect of condition (*F*_1,45_ = 18.25, *p* < 0.001, $\eta_{p}^{2}=0.29$) with participants slower to respond to targets in the threat condition (safe *M* = 306.84 ms, s.d. = 48.01 ms, threat *M* = 311.51 ms, s.d. = 45.24 ms; figure 2). Participants' RT was not affected by valence (*F*_1,45_ = 1.395, *p* *=* 0.24, $\eta_{p}^{2}=0.03$) nor was there an (valence × condition) interaction (*F*_1,45_ = 0.02, *p* = 0.89, $\eta_{p}^{2}<0.001$; see table 1 for RT to each stimulus and trial type).

Bayesian analysis confirmed that a model with main effect of condition was the winning model with BF_10_ = 122.59.

### 3.2. Study 2

#### 3.2.1. Threat of shock manipulation

Subjective ratings of anxiety levels were significantly higher during the threat condition relative to the safe condition (*t*_51_ = 6.15, *p* < 0.001, *d* *=* 0.92; safe *M* = 3.21, s.d. = 2.12; threat *M* = 5.27, s.d. = 2.34). Bayesian analyses confirmed a model with main effect of condition was the winning model, with a decisive magnitude difference (BF_10_ = 120 160).

#### 3.2.2. Accuracy

As with Study 1, we did not find a significant main effect of condition (*F*_1,54_ = 0.25, *p* = 0.62, $\;\eta_{p}^{2}<0.01$) or valence (*F*_1,54_ = 0.41, *p* = 0.52, $\;\eta_{p}^{2}<0.01$), nor was there a significant (valence × condition) interaction on participants' accuracy (*F*_1,54_ = 0.13, *p* = 0.72, $\eta_{p}^{2}<0.01$; see table 2 for response accuracy to each stimulus and trial type).

**Table 2.** Response accuracy for each stimulus and trial type for Study 2.

	mean	s.d.
*response accuracy*		
fearful ‘no-go’ distractors (threat condition)	0.39	0.25
fearful ‘no-go’ distractors (safe condition)	0.37	0.24
happy ‘no-go’ distractors (threat condition)	0.40	0.29
happy ‘no-go’ distractors (safe condition)	0.42	0.26
fearful ‘go’ targets (threat condition)	0.96	0.048
fearful ‘go’ targets (safe condition)	0.98	0.033
happy ‘go’ targets (threat condition)	0.95	0.097
happy ‘go’ targets (safe condition)	0.97	0.047
*mean RT* (*ms*)		
fearful ‘no-go’ distractors (threat condition)	271.17	87.20
fearful ‘no-go’ distractors (safe condition)	263.03	76.48
happy ‘no-go’ distractors (threat condition)	247.49	72.65
happy ‘no-go’ distractors (safe condition)	263.62	68.39
fearful ‘go’ targets (threat condition)	318.87	48.43
fearful ‘go’ targets (safe condition)	304.16	46.49
happy ‘go’ targets (threat condition)	314.01	53.19
happy ‘go’ targets (safe condition)	299.66	47.80

Bayesian analysis confirmed that no models had a BF_10_ > 1 indicating no evidence of an effect of valence or threat. Indeed, a model including an interaction term between valence and threat was substantially (166.67) worse than the null, providing evidence against our hypothesis of a threat × valence interaction.

#### 3.2.3. Reaction time

**Figure 3.**
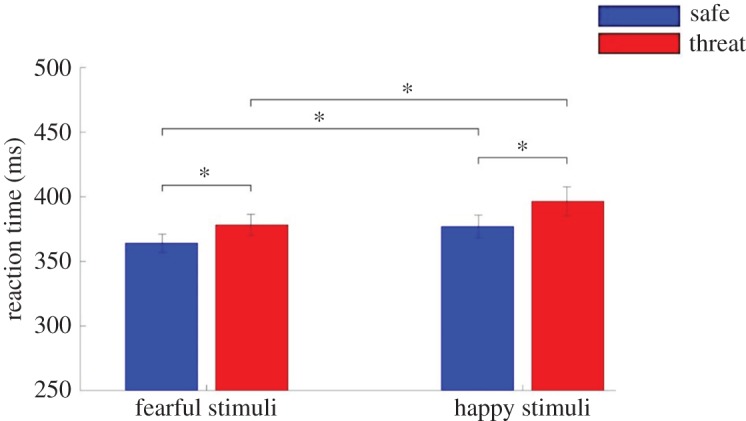
Study 2: Participants were significantly slower to respond to targets (‘go’ stimuli) when under threat of shock (*p* = 0.006) and faster to respond to fearful stimuli overall (*p* = 0.004). Error bars represent one s.e.m.

There was a main effect of condition (*F*_1,54_ = 8.78, *p* *=* 0.005, $\eta_{p}^{2}=0.14$). Participants were slower to respond to targets in the threat condition (threat *M* = 387.30 ms, s.d. = 66.69 ms; safe *M* = 370.51 ms, s.d. = 55.84 ms; figure 3). There was also a main effect of valence (*F*_1,54_ = 8.81, *p* *=* 0.004, $\eta_{p}^{2}=0.14$) with participants faster to respond to fearful targets (fearful *M* = 371.12 ms, s.d. = 53.40 ms; happy *M* = 386.70 ms, s.d. = 67.12 ms; figure 3) but no condition × valence interaction (*p* *=* 0.61; see table 2 for RT to each stimulus and trial type).

Bayesian analyses confirmed that a model including independent main effects of valence and condition was substantially (209.02) better than the null.

#### 3.2.4. Post hoc between-subject analyses

Correlations were run to look at response accuracy and response time differences (to ‘no-go’ and ‘go’ stimuli, respectively) across threat and safe conditions against STAI trait scores in both studies, and blood alcohol measures in Study 1. The correlation between trait anxiety and threat minus safe RT to fearful targets had the highest correlation in both studies 1 and 2 (*r* = 0.256, *p* = 0.188, *r* = 0.233, *p* = 0.087).

### 3.3. Follow-up colour data

As all subjects in Study 2 completed the task with the same background colours, we collected a small set of data in the laboratory with reversed colours to determine whether effects were driven by colour rather than condition. Pooling these data with that collected in Study 2, the effect of threat on RT remained, with participants slower to respond under threat of shock (*F*_1,71_ = 6.40, *p* = 0.01, $\eta_{p}^{2}=0.083$; threat *M* = 383.87 ms, s.d. = 63.35 ms; safe *M* = 369.10 ms, s.d. = 73.91 ms). Crucially, however, there was no significant interaction between colour and condition (*F*_1,71_ = 0.12, *p* *=* 0.73, $\eta_{p}^{2}=0.002$), so background colour is unlikely to be driving the effect of threat of shock.

Bayesian analysis confirmed a non-significant interaction between condition and background colour (BF_10_ = 0.998). Indeed, a model containing this interaction term was 41.36 times worse than a model including a main effect of condition, enabling us to reject the hypothesis that background colour is driving the effect of condition.

## 4. Conclusion

We demonstrate that threat of shock slows response times to ‘go’, target stimuli and in Study 2 observe that reaction times to fearful stimuli were faster relative to happy stimuli. However, there was no evidence in support of our hypothesis that threat of shock would selectively increase accuracy to task-congruent stimuli (i.e. that accuracy at withholding a response to fearful ‘no-go’ distractors would increase when under threat of shock).

Across studies, we replicated a previous finding that threat of shock slows down reaction times to target ‘go’ stimuli [9,13,24]. We suggest an explanation for this, where being cautious when under threat of shock prevents impulsive responding, and reduces the risk of harmful behaviour. This is consistent with the Pavlovian account of behavioural control in which an aversive context (such as the threat of shock condition in our study) gives rise to a ‘pre-potent’ inhibition of a behavioural response [25,26]. We propose that the faster responses to fearful faces may have resulted because these stimuli capture attention [27], and so are responded to more quickly. This distinction could potentially be assessed in future studies using a dot probe paradigm to measure attentional capture by threatening context versus fearful faces (e.g. [28,29]).

In the first study, there were no main effects of valence or improved inhibition under threat overall (and therefore no evidence to support the idea of an affective bias, i.e. a valence × condition interaction in response accuracy). Bayesian analysis confirmed that a null model was in fact 90.91 times better than a model including this interaction term. Thus, although threat of shock had an influence on affective state, demonstrated by significantly different subjective ratings of anxiety between sessions, and slower reaction times, it had no effect on response inhibition (i.e. improved accuracy at inhibiting a response to fearful faces under threat of shock). One explanation for this could be the stimulus presentation times. Here, the stimuli were presented for 250 ms, while in a previous study (which demonstrated an affective bias) the faces were presented for 1000 ms. These different presentation times across studies correspond with different average reaction times (first study safe condition = 311.21 ms, threat condition = 322.07 ms, previous study safe condition = 739 ms, threat condition = 741 ms). As it is thought that conscious processing of faces occurs above chance for presentations of 330 ms or more [30], the motor response could have been made before the valence was detected. To explore this possibility, during Study 2 the stimulus presentation times were increased to 750 ms. Here, we replicate the effect of condition on RT, with threat of shock slowing down responses, but now also observe a main effect of valence on RT, with participants faster to respond to fearful faces across conditions. This suggests that for valence effects to emerge, stimulus presentations need to be longer.

In Study 2, our results did not support our hypothesis that there would be an improvement in accuracy at withholding a response to fearful distractors under threat of shock—i.e. a condition by valence interaction. These hypotheses would be expected based on previous research demonstrating an increased response to aversive stimuli [10,14] and inhibitory control on a neutral version of this task [9,11] under threat of shock. However, we also do not see a main effect of condition or valence on accuracy in any of the present studies. This discrepancy is unexpected. One possible explanation is that the average reaction times in both conditions in Study 2 are slower relative to the original, non-affective version of the SART [9,11]. Perhaps this slowing of responses makes it easier to inhibit responses and so a smaller number of errors are made, resulting in fewer accuracy effects. However, this fails to explain why there was no such effect in Study 1. An alternative explanation, therefore, is that adding the emotional component to the SART makes it easier to detect stimuli and therefore easier to inhibit a response, resulting in no accuracy improvement under threat. As evidence of this, our average accuracy is 0.75 under threat and 0.74 under safe, whereas in the original neutral task, accuracy was 0.79 under threat and 0.70 under safe. One possibility therefore is that there was a selective impairment under safe in the original study (perhaps driven by the engagement of a habitual go response) that was improved by threat. Perhaps the presence of emotional stimuli, especially fearful faces, was enough to focus attention on the task during the safe conditions (i.e. prevent the engagement of a habitual go response) too and hence reduce the threat safe differential. A final explanation is that there might be a conflict between a pre-potent bias to respond quickly to emotional stimuli which takes precedent over task demands for inhibitory control and, as such, removes the ability for the threatening context to improve inhibition. Explicitly testing both neutral and emotional versions of this task in future work would help clarify this.

We also highlight that the effect of threat of shock on RT remained when pooling results from the second and third studies, and that background colour did not interact with condition. This indicates that the effect of shock we see is not driven by the background colour and is consistent with prior research that has also demonstrated no influence of background colour on the effects of threat of shock [31,32].

It should be noted that our paradigm, which uses a threat of shock manipulation, attempts to exploit anxiety and not fear responses. Fear can be thought of as a short lasting response to a predictable, threatening stimulus, whereas anxiety can be thought of as a response to unpredictable and prolonged threats. These different responses are behaviourally, neurally and pharmacologically discrete [33–35]. However, there is a large body of work on fear generalization, in which fear responses can transfer across stimuli. As a caveat, therefore, we recognize that it may be possible that threat-related behaviour spills over into the safe condition, in keeping with a large body of research on fear conditioning and threat generalization in anxiety [36,37]. Future work could test this using between-subject designs. In addition, participants are told that they will only receive a shock in the threat condition. We do not believe there is potential for uncertainty or distrust, but future research could also assess this directly using self-report trust measures. Finally, whilst it is possible that the that fearful face stimuli could prime generalization behaviour, it should be noted that subjective ratings of anxiety were still significantly higher in the threat condition relative to the safe condition.

It should be noted that the STAI measure we used to determine dispositional anxiety may not be the best measure to assess anxiety related to social stimuli such as faces. Future work may seek to determine individual differences in social anxiety. We also highlight the somewhat unique data collection environments which may have impacted on the data. Data were collected in very busy rooms with event attendees free to wander around and watch the testing. After optimization of the task in Study 2, we observed a main effect of valence, which suggests that participants were concentrating on the task. However, attention on the task might plausibly be less than it would have been in a controlled laboratory setting. This could be particularly relevant as individual differences in attentional control mechanisms interact with performance across threat and safe conditions [12] and prominent theories of anxiety [38,39]. However, the lack of a study effect in the pooled data counteracts this hypothesis, as there was no differential performance in the lab-collected data. It is also worth mentioning that alcohol was widely available at the first event. In order to address this potential confound and to also look at potential interactions between anxiety level, blood alcohol concentration and task performance, blood alcohol measurements were recorded. Whilst blood alcohol concentration did not correlate with any performance measure (*p* > 0.05) it should be noted that the breathalyser measures may have been unreliable. Since alcohol reduces anxiety and the key to investigating this affective bias in our task is comparison of performance across safe and threat (stress-inducing) conditions, the presence of unaccounted for alcohol-driven variance might explain our null finding on the first event. Nonetheless, unlike traditional controlled laboratory settings, these results can be considered a naturalistic representation of performance during a social event and demonstrate collecting data during public engagement events is possible. Indeed, we were especially keen to publish this data as part of this continuing engagement process such that participants will eventually be able to track their involvement all the way to this paper.

To summarize, across two studies we replicated a previously observed effect, namely that threat of shock reliably slows down reaction times for ‘go’ responses [9,24,26], and, when subjects are given longer to respond in Study 2, show that fearful faces are responded to faster than happy faces [14]. Our data do not, however, support the hypothesis of a threat by valence interaction in accuracy, nor do we see an overall improvement in accuracy during induced anxiety, potentially because the presence of emotional stimuli increased the anxiogenic nature of the safe condition.

## Supplementary Material

Threat minus safe reaction time and accuracy vs trait anxiety scores.

## Supplementary Material

Threat minus safe reaction time and accuracy vs trait anxiety scores.

## Footnotes

1 For event programme, see here: http://www.rigb.org/docs/questioning_reality_floorplan_web_0.pdf.

2 For event programme, see here: https://wellcomecollection.org/sites/default/files/Friday%20Late%20Spectacular%20Feeling%20Emotional%20Programme_0.pdf.

## Data accessibility

The data have been deposited in an external repository: (http://dx.doi.org/10.6084/m9.figshare.1609723). See the electronic supplementary material for correlational graphs (http://dx.doi.org/10.6084/m9.figshare.4986614).
