# Supplementary figures and images for: The impact of induced anxiety on affective response inhibition
Source: R Soc Open Sci. 2017 Jun 7;4(6):170084. doi: 10.1098/rsos.170084 (PMC5493909; doi:10.1098/rsos.170084)

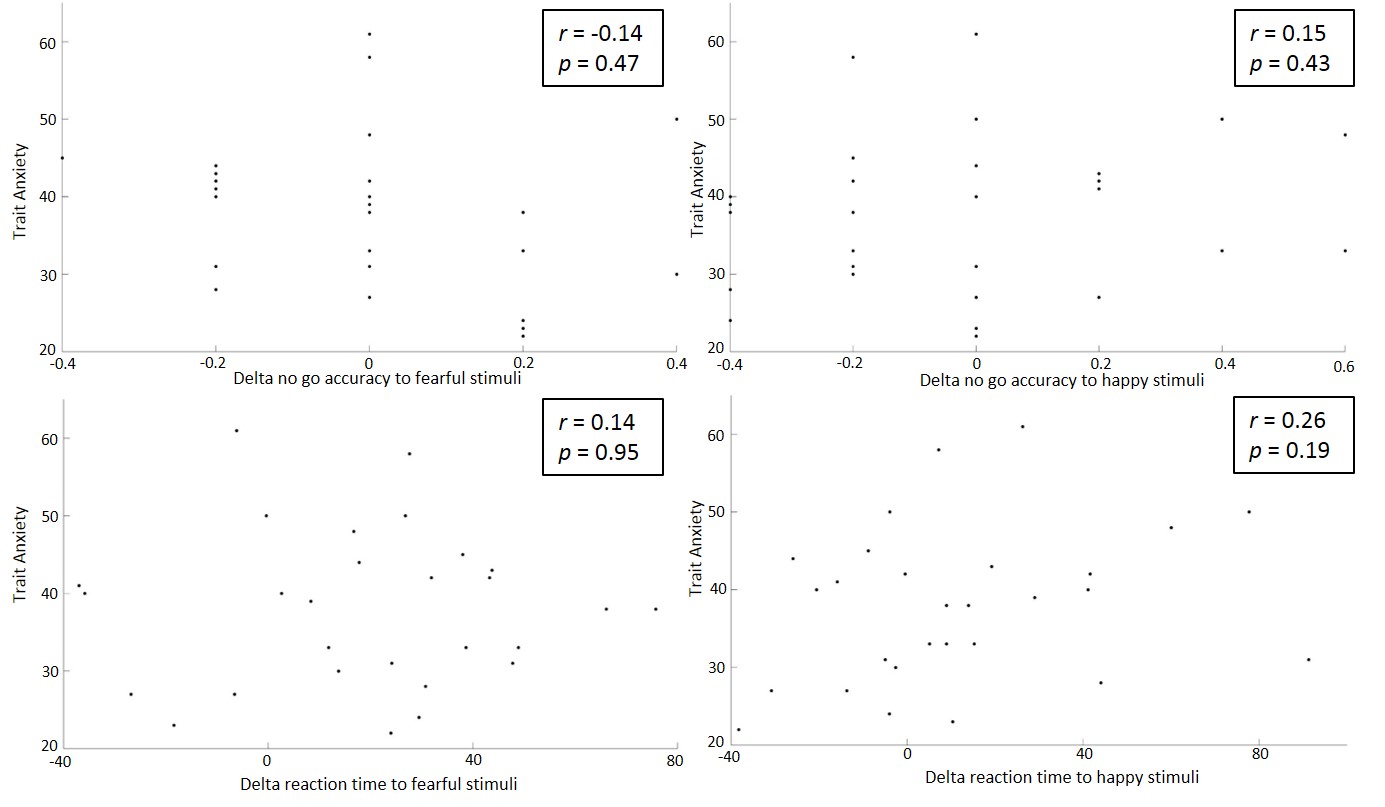

Supplement: Threat minus safe reaction time and accuracy vs trait anxiety scores. [file rsos170084supp1.jpg]

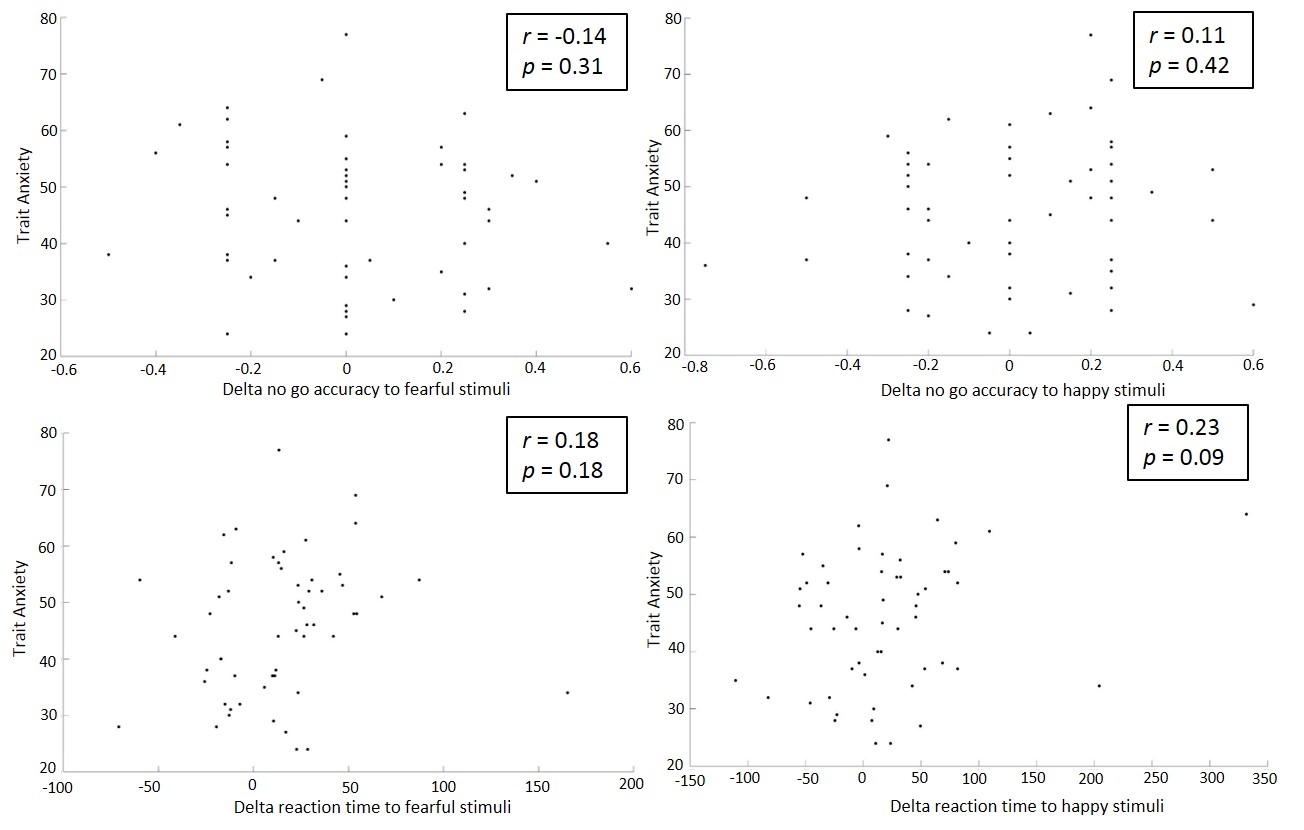

Supplement: Threat minus safe reaction time and accuracy vs trait anxiety scores. [file rsos170084supp2.jpg]
